# The Use of Fiber Bragg Grating Sensors in Biomechanics and Rehabilitation Applications: The State-of-the-Art and Ongoing Research Topics

**DOI:** 10.3390/s121012890

**Published:** 2012-09-25

**Authors:** Ebrahim Al-Fakih, Noor Azuan Abu Osman, Faisal Rafiq Mahamd Adikan

**Affiliations:** 1 Center for Applied Biomechanics, Department of Biomedical Engineering, Faculty of Engineering, University of Malaya, 50603 Kuala Lumpur, Malaysia; E-Mail: engr.fakih@yahoo.com; 2 Photonics Research Group, Department of Electrical Engineering, Faculty of Engineering, University of Malaya, 50603 Kuala Lumpur, Malaysia; E-Mail: rafiq@um.edu.my

**Keywords:** fiber Bragg grating, Bragg sensors, biomechanics, rehabilitation

## Abstract

In recent years, fiber Bragg gratings (FBGs) are becoming increasingly attractive for sensing applications in biomechanics and rehabilitation engineering due to their advantageous properties like small size, light weight, biocompatibility, chemical inertness, multiplexing capability and immunity to electromagnetic interference (EMI). They also offer a high-performance alternative to conventional technologies, either for measuring a variety of physical parameters or for performing high-sensitivity biochemical analysis. FBG-based sensors demonstrated their feasibility for specific sensing applications in aeronautic, automotive, civil engineering structure monitoring and undersea oil exploration; however, their use in the field of biomechanics and rehabilitation applications is very recent and its practicality for full-scale implementation has not yet been fully established. They could be used for detecting strain in bones, pressure mapping in orthopaedic joints, stresses in intervertebral discs, chest wall deformation, pressure distribution in Human Machine Interfaces (HMIs), forces induced by tendons and ligaments, angles between body segments during gait, and many others in dental biomechanics. This article aims to provide a comprehensive overview of all the possible applications of FBG sensing technology in biomechanics and rehabilitation and the status of ongoing researches up-to-date all over the world, demonstrating the FBG advances over other existing technologies.

## 1. Introduction

In the last decade, fiber Bragg gratings (FBGs) have shown a great potential for applications in the field of biomechanics and rehabilitation engineering due to their prominent advantages such as their small size, biocompatibility, chemical inertness, immunity to electromagnetic interference (EMI) and multiplexing capability [1,2]. These characteristics make FBGs suitable for human body uses that adapt to the sensor material so that they can be used for *in vivo* measurement and can be left for long-term monitoring [3]. They also offer high-performance alternative, in comparison to standard technologies like electrical strain gauge (ESG), piezoelectric, resistive or other solid-state sensing, either for measuring physical parameters or for performing high-sensitivity biochemical analysis [4].

Presently, FBGs are widely used for specific applications in aeronautics, the automotive industry, structure monitoring in civil engineering and undersea oil exploration [5–7]; in order to measure various parameters such as strain [8], force [9], pressure [10], displacement [11], temperature [12], humidity [13] and radiation dose [14]. On the other hand, most studies towards synergizing optical FBG sensing technology with biomechanics and rehabilitation applications are very recent [3] and have not been commercialized yet. They have been demonstrated for measurement of a wide variety of parameters; including strain inside and on the surface of intact and plated bones, shrinkage stresses in bone cement during polymerization, pressure mapping in orthopaedic joints, stresses in intervertebral discs, deformation in chest wall to study lung biomechanics, pressure distribution in Human Machine Interfaces (HMIs), forces induced by tendons and ligaments, angles between body segments during gait, and many others in dental biomechanics.

To the best of our knowledge, only two articles have partially discussed the general applications of FBG sensing technology in biomechanics [3,15], however, they both did not fully cover all the previously-reported and the very recent and emerging applications of FBGs in the field of biomechanics and rehabilitation engineering. This article aims to provide a comprehensive overview of the applications of FBG technology in biomechanics and rehabilitation in terms of the very recent progress and the status of ongoing researches up-to-date all over the globe. Furthermore, a comparison between FBGs and other conventional technologies is also presented in order to show that FBG technology demonstrates greater feasibility for most of applications in this field.

## 2. FBG Working Principles

**Figure 1.**
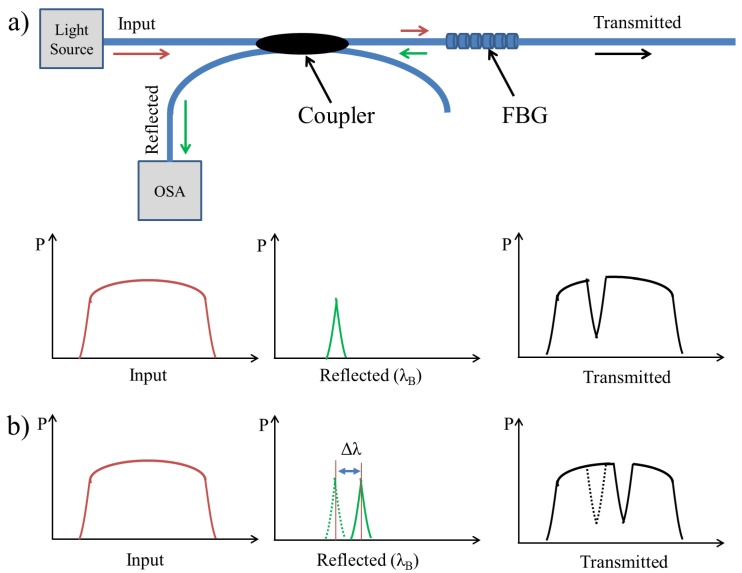
(**a**) The light source (Brown color) is transmitted through the FBG and a narrow band (Green color) is back-reflected, centred around *λ*_B_, and monitored by OSA. (**b**) The back-reflected band is shifted (Δ*λ*) shortly after applying external perturbations.

The FBG involves a spatially periodic modulation of the refractive index along specific region of a fiber's core written in a short segment of a single mode optical fiber with a cladding diameter of 125 μm [16–18]. If light from a broadband source is coupled into an optical fiber containing the FBG, a narrow spectrum is back-reflected and centred around the so-called Bragg wavelength *λ*_B_ (Figure 1(a)) [19–22] which depends on the periodic variation *Λ* of the FBG and the effective refractive index *n*_eff_ :

(1) $$\lambda_{\text{B}}=2n_{\text{eff}}\Lambda$$

When the FBG sensor is subjected to external mechanical or thermal perturbations, the back-reflected peak wavelength will be shifted (Figure 1(b)) according to the extent to which the external perturbations influence the FBG sensor that is spectrally very sensitive when subjected to axial strain [7,23–25]. The axial strain affects the FBG response directly through the compression and expansion changes in the spacing of the periodic variation, *Λ*, and through the strain-optic effect which induces a change in the effective index of the optical fiber, *n*_eff_ [2,17,18,20]. The temperature increment Δ*T* also affects the refractive index and the grating period, resulting in a change in *λ*_B_ [22]. The influence of strain and ambient temperature on the grating period and the refractive index can be expressed as follows:

(2) $$\Delta \lambda_{B}=2\left[\Lambda \frac{\partial n}{\partial l}+n\frac{\partial \Lambda }{\partial l}\right]\Delta l+2\left[\Lambda \frac{\partial n}{\partial T}+n\frac{\partial \Lambda }{\partial T}\right]\Delta T$$

where Δ*l* is the change in FBG length caused by strain and Δ*T* is the temperature change. To determine the Bragg wavelength *λ*_B_, optical spectrum analyzers (OSA) are commonly used when doing experiments inside laboratories, and, for experiments conducted outside, other specialized interrogators are employed. With high-speed interrogation systems, the FBG sensors would be more suitable for monitoring strains in locations subjected to dynamic loads [20].

## 3. Applications

### 3.1. Bone

Knowledge of the biomechanical behaviour of the musculoskeletal system is crucial in understanding bone diseases and in designing medical devices [26]. The skeleton normally adapts to mechanical loading, thus understanding of how bone deformation under load occurs is of interest. Several methods have been proposed for the measurement of bone deformation under load. The strain gauge (SG) was the gold standard whose electrical resistance varies proportionally to the amount of strain. A research group based in Israel used SGs for *in vivo* measurement of strain in human bone in six subjects during running, stationary bicycling, leg presses and stepping, using bone staples made of electrical SGs [27,28]. Recently, several researchers have studied the potential of FBG sensors for *in vivo* applications as a measurement tool for bone strain, concluding that FBGs have shown competitive advantages over the conventionally-used SGs. FBGs are completely made of biocompatible silicate (SiO_2_) glass ceramics materials [26,29] which have a smaller risk for infection, whereas the SG itself represents a foreign body implant. They can also be used in locations where the use of conventional SGs is technically inapplicable [3,26]. For example, when an SG is loaded, its electrical resistance varies proportionally to the applied strain; therefore, it cannot be used in an electromagnetic environment. Moreover, there are other distinguishing advantages of FBGs when compared to the SGs: they can easily adhere to bone and other irregular surfaces [26].

**Figure 2.**
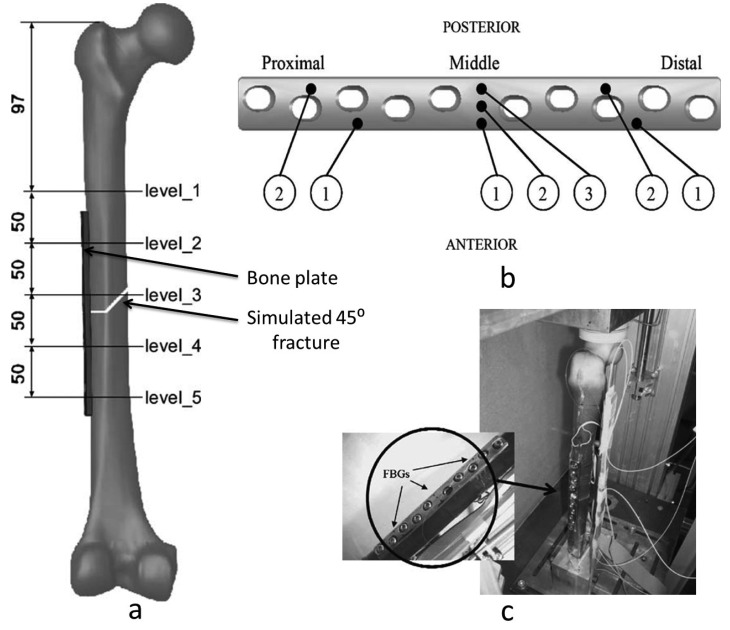
(**a**) The simulated 45° fracture and the locations of the SGs on the intact and plated synthetic Femurs. (**b**) The locations of the FBGs on the bone plate. (**c**) Representation of the experimental apparatus used and detail of bone plate with FBGs [30].

Talaia *et al.* [30] have determined bone strains in plated and intact synthetic femurs using FBGs and SGs in order to study the effects of fracture fixation plates. For the intact femur, 20 axial SGs were placed on the anterior, posterior, medial and lateral sides at different levels of cortex femur. A simulated 45° fracture on the other femur sample was made and fixed using a 10-hole stainless steel bone plate (Figure 2(a)). Seven FBGs were glued at different locations along the bone plate (Figure 2(b)) and 13 SGs were fixed to the cortex femur (Figure 2(c)). A static load of 600 N was applied to both the plated and intact synthetic femurs and bone strains were recorded at the same sites on the cortex of the femurs and on the bone plate. The sensors have shown good linearity for the different loads. However, FBGs made it much easier to measure strain on bone plates than with the conventional SGs and were more feasible to monitor the evolution of the strain in bone. From the biological point of view, FBGs are able to assess the stiffness of callus formation of fractured bones.

Fresvig *et al.* [26] have evaluated the use of FBG sensors to detect deformation in human cadaver femur bone under *in vitro* loading conditions, with potential *in vivo* application. An acryl tube and a human femoral bone sample of the same length were used in this study. Each sample was instrumented with four polyamide coated FBG sensors and four SGs, interspersed at every 45°, which were placed around the circumference at the central part of each sample. The two samples were loaded using a Mechanical Testing Machine and the induced strains were recorded accordingly. The FBG methodology showed its suitability for dynamic *in vitro* measurements. Generally, both types of sensors, FBGs and SGs, produced no significant differences, suggesting that the FBG sensors could be successfully implemented for *in vivo* measurements.

**Figure 3.**
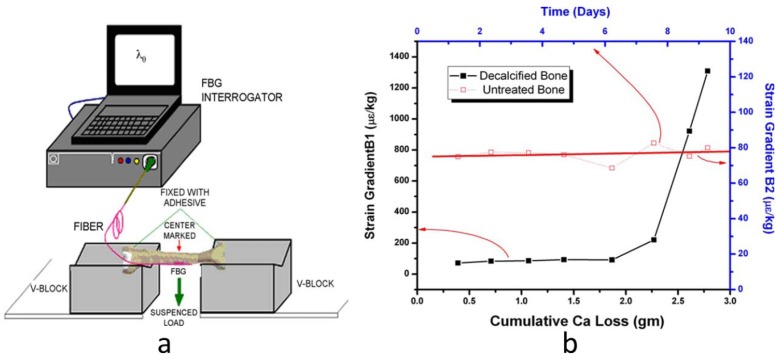
(**a**) The schematic of experimental set-up. (**b**) The comparison between strain response of decalcified and untreated bones [31].

FBG sensors can also be used to understand to what extent the bone calcium loss affects strain response of bone [31]. Two samples of goat tibiae were used; one decalcified in steps while the other remained untreated for a comparative study. Two FBG sensors were directly attached at the midpoint of both samples. Thereafter the two samples were stressed with loads ranging from 0.1 kg to 4 kg using a three-point bending test as shown in Figure 3(a). The strain values were calculated from the FBG wavelength shifts that had been recorded for both samples at the same time in order to understand the effect of decalcification and that of degeneration with time. The strain generated for the untreated sample remained largely unchanged during the experiment, while for the decalcified sample; strain values for same loads increased and became much higher with more calcium loss (Figure 3(b)). By using FBG technique, the strain response gives a direct indication of the degree of calcium present in bone.

All the above-mentioned studies suggested that FBG sensors have a potential to be used as a novel approach to assess bone biomechanics *in vivo*. Recently, Carvalho *et al.* [32] investigated the sensing capability of FBGs when placed in direct contact with human osteoblast cells cultured around the FBG fiber and assessed the reaction of these cells to optical fibers. It was found that osteoblasts exhibited a great capability to adhere and grow over the fiber and the protective polymer coating as well. Furthermore, the FBG sensing capability was not affected throughout the culture period. Hence, these excellent cytocompatibility and sensing capability of optical FBG fibers suggest the possibility of its osseointegration anticipating a variety of *in vivo* applications in bone mechanical dynamics.

Despite the great ability of bone tissues to adapt to optical fibers, enabling the use of FBG sensors for *in vivo* applications, there are some obstacles still hampering their use for full-scale implementation in this field. The need of fiber link between the FBG sensing area in the vicinity of patient and the measuring unit is the most common problem [3,15]. In hospitals or mobile care units where this drawback is minimized, portable interrogators could be employed, but for continuous monitoring of patients day-to-day activities, which is an important requirement for orthopaedic treatments and biomechanical studies, these devices can be cumbersome for patients [3,15]. It is possible with today's technology to produce very small wearable interrogators in the near future with no external fiber links, opening up the evolutionary road towards effective *in vivo* applications of FBG sensors in human body.

**Figure 4.**
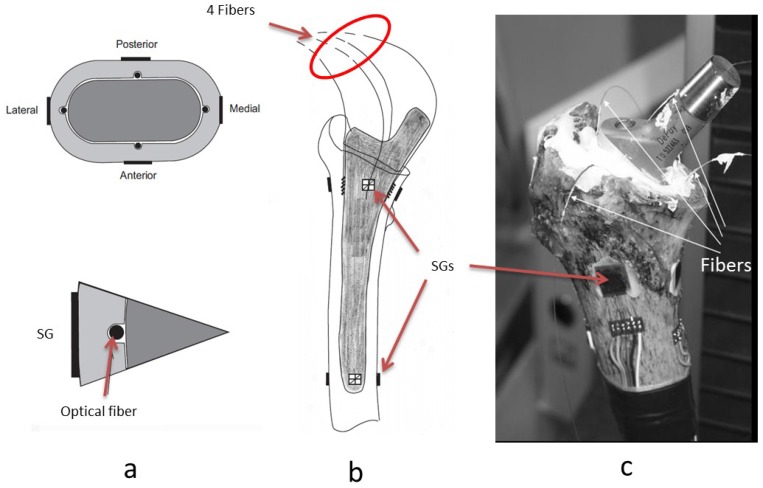
Schematic of the experimental set-up. (**a**) A cross-section of the implanted femur, showing the locations of FBGs and SGs. (**b**) Schematic drawing of the femur with cemented prosthesis, showing the internal (optical fibers) and external (SGs) positions of sensors at the proximal and distal levels. (**c**) Image of the experimental set-up of an implanted cadaveric femur [33].

In issues pertaining to the influence of total joint replacements on bone strains, a very recent study by Reikeras *et al.* [33] investigated the differences between internal and external cortical strains in the proximal femur after insertion of a cemented and uncemented hip stem prosthesis using SGs and FBGs. One cadaveric femur was removed within 24 hours from a 64 years old male patient who died from a heart attack. Four SGs were attached at the external femoral cortex both proximally and distally and four optical fibers were sealed at the internal femoral cortex proximally, as shown in Figure 4. When axial load was applied onto the prosthesis, the strains at the proximal level were simultaneously recorded at the internal cortex (by FBGs) and at the external cortex (by SGs) and were then compared in order to evaluate the difference in strain patterns at the internal and external cortices. Previously, it had been assumed that strain on the external cortex reflects that at the internal cortex. However, this study showed that the strain pattern on external cortex was significantly different from that obtained at the internal cortex. In other words, the situation on the external cortex does not correspond to that at the internal cortex and the strain at internal cortex cannot be deducted from strain measured at the external cortex. The ingrowth of bone and stability of the prosthesis depend on the internal cortical surface, therefore, future studies should pay attention to this fact.

**Figure 5.**
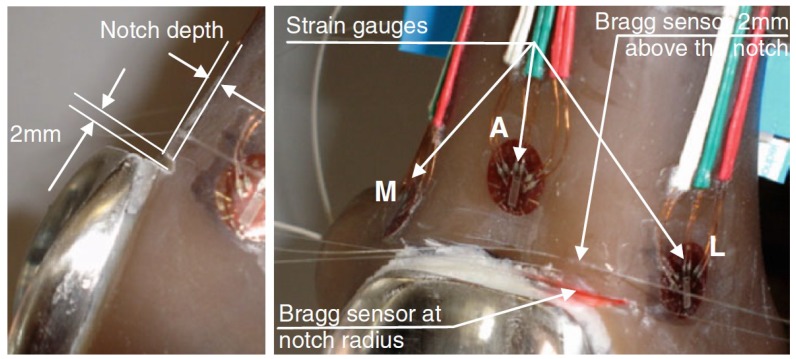
Images depicting the composite femur models with notch. (**left**) The notch dimensions, and (**right**) the positions of SGs (A anterior, M medial, L lateral) and FBGs [34].

The strain behaviour at the anterior femoral notching region during total knee replacement and its consequences on the cortex of the distal femur was studied by Completo *et al.* [34]. They also questioned the effect of using femoral stem in an anterior femoral notching to reduce the fracture risk. It had been hypothesized that femoral notching weakens the cortex of femur which can make it susceptible to fracture in the early postoperative period [35]. In this study, 12 synthetic femurs were selected and undergone experiments under two load scenarios. Femoral components with and without femoral stems were implanted in femurs with different notch sizes to predict experimentally the strain levels at the notch edge using FBGs and at notch region using SGs (Figure 5). The most important finding of this study is that the strain behaviour was dissimilar for the different notch depths. For notch depths lower than 5 mm, the use of stem reduces the strain level at the notch edge to values below the intact femur condition, while for depths greater or equal to 5 mm, the strain levels at the notch edge were higher than the intact femur condition (ranging from +10 to +189%). This suggests the use of a prophylactic stem for notch depths greater than 5 mm, contributing to reducing the fracture risk.

### 3.2. Bone Cement

Damage in bone cement induced by long-term dynamic loading that causes prosthesis loosening is one of the main reasons for total hip replacement failures [36]. *In vitro* studies using human and animal bones and models could be useful to verify the action of the cement, both during the curing process and after consolidation. Embedded rosette gauges for strain measurement of bone cement in femoral prosthesis had been previously used by Stolk *et al.* [37]. However, the FBGs have shown their advantages in studies pertaining to these issues. Ramos *et al.* [38] assessed the accurate stress and strains magnitudes inside cement mantles using FBG sensors which could be useful to predict the mechanical failure. Twelve FBG sensors were bonded to a pre-designed femoral prosthesis; four at the proximal, distal, and half of the stem, and were strategically attached in the anterior, posterior, medial and lateral aspects. The FBG-instrumented prosthesis was placed inside the femur canal in a way the FBGs were placed at a distance of 1 mm from the cement-implant interface. The cement strains measurement using this technique is less time-consuming and easier to implement than the one using SGs.

FBGs can help predict the functional durability of PMMA. Frias *et al.* [39] tested the capability of FBGs to measure strains inside bone cement during different mechanical tests at real-time. Bone cement was tested at different temperatures and load conditions according with those expected inside the human body. The mechanical behaviour of different specimens of PMMA bone cement was studied in different short-term quasi-static tests to predict the PMMA durability. Different test procedures allowed for measuring the tensile and compression strains on PMMA bone cement using FBG sensors. The embedded FBG was subjected in independent tests to some of the physical parameters that bone cement is simultaneous submit in an artificial joint. The optical reflected spectrum was recorded at the end of each test using an OSA. All the strain measurements inside bone cement reported a linear response that shows a good adaptation and optimization of the load transfer between the PMMA cement and the embedded FBG sensor.

### 3.3. Orthopaedic Joints

An embedded array of FBGs exhibited its feasibility to be used at orthopaedic joints interfaces for pressure mapping and the measurement of contact forces and stresses. It is necessary for those involved in prostheses research and biomechanics studies to understand the contact area and contact stress at Orthopaedic joints as well as the alignment of the joint interface (*i.e.*, tibiofemoral interface). Measurements of contact area, contact force and stress distribution in the tibiofemoral joint have been made using commercially available stress-sensitive films such as I-Scan and Fujifilm [40–43]. However, the biomechanics of knee joint might be altered after the insertion of these films at the articulating surfaces.

Due to their flexibility and multiplexing capability, FBG sensors have shown an edge over these conventional measurement systems as they are suitable to be used in any irregular-shaped interface surfaces (*i.e.*, knee joint) and, in some cases, they do not change the natural contact topology of joint capsule (*i.e.*, hip joint). The application of an FBG sensor for pressure mapping at the prosthetic knee joints was first reported by Mohanty *et al.* [44,45]. In these cadaver studies, a rectangular array of FBGs was embedded into a tibial spacer that was inserted at the tibiofemoral interface and the changes in the contact stress distribution caused by joint misalignment in extension and flexion were monitored. This spacer sensor can be used to correct malalignment during total knee arthroplasty. It can be used *in vitro* for studying the biomechanics of the prosthetic knee joint especially for curved and conforming designs.

The commercial sensitive films were also used for stress measurement in hip joint [46], but their application is severely limited by the amount of dissection required to insert them in this joint. This insertion approach is considered to be too interventional when used in the natural hip [47]. FBG sensors have shown their great potential to overcome some of the sensitive films limitations because they have key advantages that make it more attractive for application in the hip joints such as the small size, mechanical compliance and biocompatibility.

**Figure 6.**
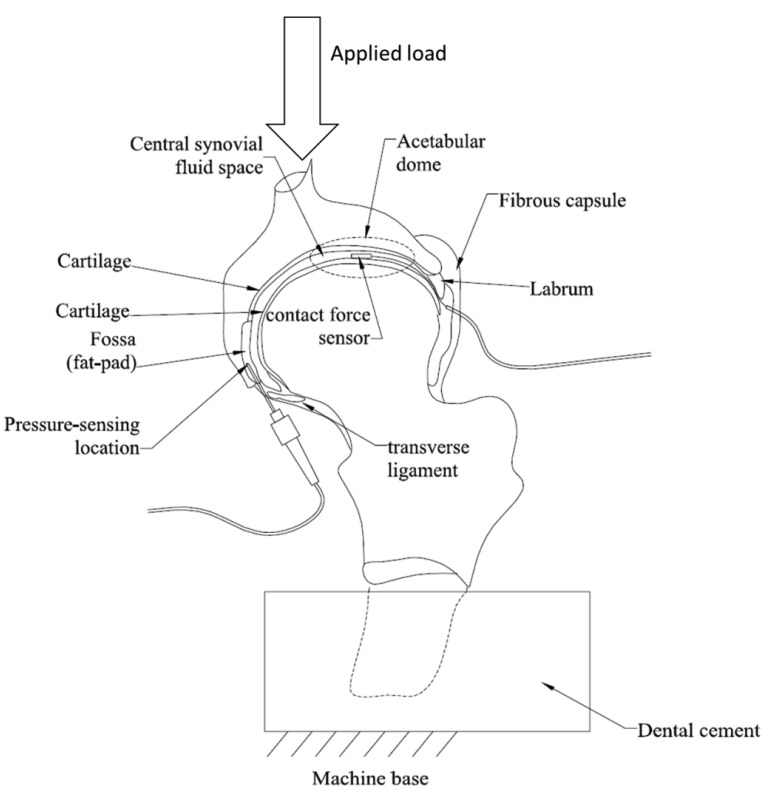
Schematic drawing of the cross-section of hip joint showing the insertion of the pressure and contact stress sensors into their optimal sensing locations. The hip stem was embedded in dental cement for fixation purposes during applying vertical loads [48].

Dennison *et al.* [48] were the first to report the simultaneous measurement of contact force/stress and fluid pressure in intact cadaveric human hips using Bragg-based sensors, in order to address the limitations shown by the conventionally-used sensitive films. Two 1 mm long uniform FBGs were inscribed in a core of two separate SMF-28 fibers, and were each coated by two layers of polymer annulus and a polyimide sheath, respectively. The polymer layer was placed between the outer diameter of the optical fiber and the inner diameter of the polyimide sheath. Two cadaveric hip samples were obtained and all musculature of the samples was dissected, but the joint capsule was left intact. The FBG contact sensor was placed in the hip joint between the femoral head and the acetabulum cup, while the pressure sensor was put in the fossa which is the active pressure sensing area (Figure 6). Numerical models were used to predict the sensor performance and the predicted sensitivity was verified through experimental calibrations. The results show that contact force and pressure measurements using FBG sensors in the hip joints exhibited repeatability and the polyimide sheath increased the sensitivity of the contact force sensor, when compared to the unsheathed sensor of the same diameter. These sensors could potentially be used to study the correlations between contact forces and pressures in healthy and deteriorated joints.

### 3.4. Intervertebral Disc (IVD)

Pressure distribution at the IVD is a key to understand the IVD biomechanics which were previously measured both *in vivo* [49,50] and *ex vivo* [51–54]. Needle-mounted SG sensors have been employed for the measurement of disc pressure [55–59] ranging over 3 MPa in cadaveric human discs [52], but it was found that these sensors show key limitations hampering their use research area. They are housed in large and rigid needles (over 1 mm diameter [60]), which when inserted cause injuries to the disc annular fibres and alter the mechanics of the intervertebral disc [61]. On the other hand, FBG strain sensors have shown excellent ability to address some of the above mentioned limitations as they are smaller in size (less than 0.5 mm) and biocompatible. Due to their small size, they can minimize the injury to the fibers of the disc annulus and the effects on disc mechanics [62]. In other words, using FBGs for pressure measurement is less invasive and disruptive than current measuring systems.

Methods to develop intervertebral pressure sensor using FBGs were first reported by Dennison *et al.* [63]. A bare FBG sensor (10 mm length and 125 μm diameter) was inserted in a hub of a 25 gauge hypodermic needle which its tip was then passed through the disc annulus so as to be placed approximately at the centre of the nucleus. The sensor was then advanced past the needle tip so that its entire length was exposed to the nucleus pulposus and the needle was left in the annulus to shield the fiber optic cable from any traction. Each lumbar spinal unit was loaded from 0 N to 2,000 N to 0 N at 10 N/s. The sensor performance was investigated in five two-vertebra human cadaveric spinal units. After each test with FBG sensor and for comparison purposes, the tests were repeated by using two SGs instead. Although the pressure measurements using FBG sensors were in agreement with those obtained in previous disc pressure studies, they were not always consistent with measurements made using current standard SGs sensors. That is because bare FBG measurements have shown poor pressure sensitivity, resulting in highly discretized data over the 3 MPa pressure ranges. These drawbacks might come from the nucleus material inhomogeneity and from the length of the FBG (over the 10 mm) which would interrupt pressure measurements. Improving the sensor design and sensitivity and its spatial resolution is very important for obtaining better measurement results.

**Figure 7.**
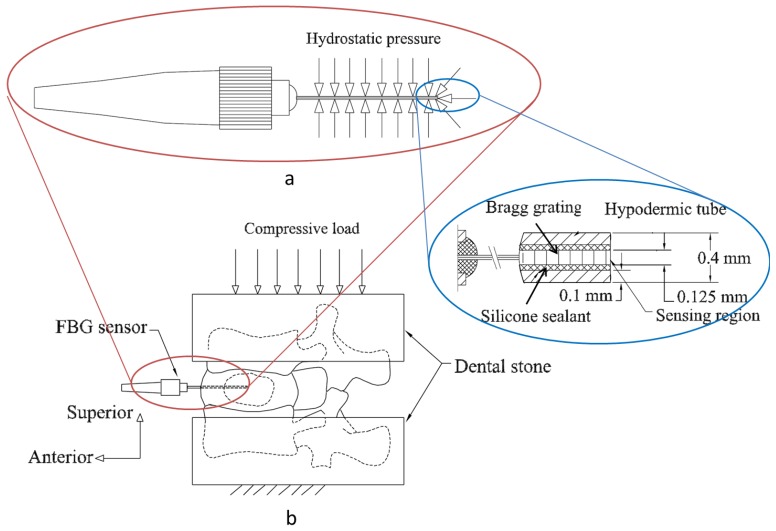
(**a**) Schematic showing the construction of the IVD pressure sensor. The hydrostatic pressure over the hypodermic tube and at the sensing region whose dimensions are shown (right inset). (**b**) Compressive loads applied to a functional spine unit embedded between dental stones and the pressure sensor inserted into the annulus [1].

The same research group have designed and constructed a new patented FBG based IVD pressure sensor [64] that has both improved pressure sensitivity (seven times greater than that of a bare fiber while maintaining extremely small size) and spatial resolution by limiting the sensing region to the tip of the sensing hypodermic tube [1]. A 10 mm length FBG was written in a single mode fiber that was housed within 50 mm stainless steel hypodermic tube so that the FBG is positioned at the end of the tube (Figure 7(a)). A silicone sealant was used to fill up the annular volume between the inner diameter of the hypodermic tube and the outer diameter of the FBG fiber in order to enhance the sensor sensitivity. The sensor tip was introduced into the nucleus center of a cadaveric porcine disc and compressive loads (from 0 N to 500 N at 40 N/s) were applied onto the superior vertebra of the spinal unit (Figure 7(b)). As applied pressure onto the hydrostatic tube is increased from 0 MPa to 3 MPa, the silicone sealant and the optical fiber deflect mainly in the FBG longitudinal axis (compressive strains), with the highest magnitude deflections occurring at the probe tip (the inset of Figure 7(a)). The deflections in the longitudinal axis decrease in magnitude along the sensor (from right to left in Figure 7(a)), and are at their minimum at the left-hand side of the sensor. The same procedures were subsequently repeated using a needle-mounted SG sensor. The measurements obtained using FBG sensor were compared to those made with the widely-used SG sensors. The FBG sensor exhibited higher sensitivity, excellent repeatability and was in agreement to those obtained using SG sensors. It is worth mentioning that this hypodermic tube containing the FBG does not have a cutting tip to ease the insertion into the disc annulus. That is why another cutting tip needle is needed to pierce the annulus prior to insertion. Future design must take this issue into account so as to dispense with the aid of the needle by constructing new FBG sensors with their built-in cutting needle.

The performance of the latter FBG sensor was validated in a subsequent study conducted by Dennison, Wild, Dvorak, Wilson and Cripton [62]. This study assessed the sensor sensitivity, linearity, and hysteresis and determined the sensor accuracy in pressure measurements of IVD as a function of compressive load in six porcine cadaveric spinal units obtained from two lumbar spines. After calibration and preconditioning, the FBG sensor was introduced into the annulus and compressive loads were applied to the functional spinal unit using a saw-tooth loading profile for both loading and unloading phases. The hydrostatic pressure at the disc annulus was monitored using the FBG sensor. Thereafter, the FBG sensor was replaced by SG sensor and same test procedures were conducted. The results of the FBG sensor were still in agreement with those obtained using SG sensors. But the FBG sensor also exhibited higher sensitivity and increased spatial resolution because its active sensing region was confined to the needle tip. What is superior with this FBG sensor over the previous disc pressure sensors is that it did not interfere with vertebral endplates and did not alter the disc biomechanics and structure. In addition, this FBG sensor is 83% smaller and more flexible than any previously-reported disc pressure sensor and can be used to make less invasive measurements of disc pressure *in vivo* and during discography.

A very recent study by Roriz *et al.* [65] has proposed an FBG sensor to measure strain due to bulging of the IVD under axial compression. This study introduced the least invasive sensor up-to-date as the needle was used only for guiding and placing the sensor and could be then removed leaving the sensor in direct contact with the disc material. A 2 mm length FBG was inserted into the disc center of an *ex vivo* porcine dorsal spinal unit using a 25 gauge hypodermic needle that was removed after placing the sensor and axial compression loads were applied causing strain in the FBG fiber which, combined with strain due to lateral disc bulging, induced changes in the Bragg wavelength. The results verified that the sensor is capable of measuring strain due to the applied loads and the bulging of the annulus fibrosus as a consequence of axial compression. However, this sensor has shown a limitation. Since the FBG fiber is stiffer than the IVD, the fiber will resist disc bulging and fail before maximum disc elongation. Therefore, this technique is not suitable for measurement of tissue physiologic strain.

These new FBG sensors can potentially increase our understanding of the correlations between the internal disc pressure and the degree of disc degeneration. These correlations may increase our knowledge of disc biomechanics and potential disc injuries and degeneration. Further studies are needed to address the effect of the sensor insertion into the disc on the load transfer and the pressure measurements.

### 3.5. Dental Biomechanics

**Figure 8.**
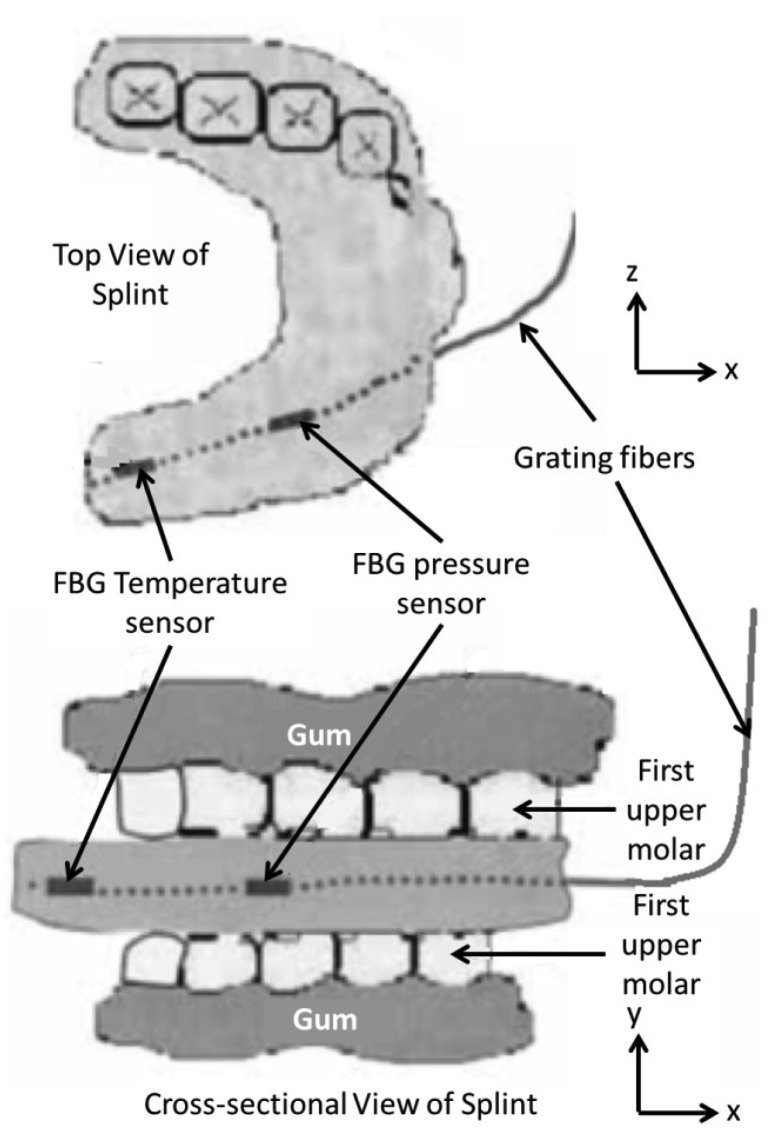
Schematic diagram of dental splint with FBG embedded sensors [66].

The application of FBG sensors in the area of dental biomechanics had been investigated earlier. Tjin *et al.* [66] have reported the first application of FBG sensors to monitor the force and temperature as a function of time in dental splints worn by patients suffering from sleep apnoea. Due to the small size of FBG sensors, it is much easier to embed them within the splint while maintaining the splint efficiency unaltered. These sensors can determine the correct use of splints when worn by the patient. Two pre-calibrated FBGs inscribed in a single strand of an optical fibre were used; one embedded within three layers of glass fibre composite materials to insulate the FBG pressure sensor from temperature effect and the other bare FBG for temperature sensing. The sensors were carefully embedded into the patient's dental splint; the pressure sensor located at the first molar and the temperature sensor located further in, where there is minimum contact with the gums (Figure 8). As the splint is being positioned in the patient's mouth, the results show an increase in both temperature and force. The advantages of these sensors, besides their effectiveness in monitoring the proper usage of the dental splints, are that they are unnoticeable due to their small size and multiplexing capability, and inherently safer than the conventionally used methods (*i.e.*, thermistors and piezo-electric pressure sensor) due to their immunity to EMI.

Another important application of FBGs in dental biomechanics is to measure the properties of dental materials like the stress-strain pattern. A few studies were reported on the possibility to use FBG sensors to study dental materials such as resin-based composites and gypsum products. FBGs can be used to measure the hardening of dental cement and the corresponding stress build-up and the volumetric shrinkage of the resin cement during polymerization [67–69]. Milczewski *et al.* [70] have used FBGs to determine the polymerization contraction and setting expansion of three types of gypsum products (plaster, dental stone, and high strength dental stone) and to monitor the strain evolution and temperature during the drying phase of the material. Two FBG sensors were used; one sensitive to the strain and temperature variations during setting, and the other placed in a double needle to monitor only the temperature variations during the reaction. The results show that the plaster of Paris demonstrated higher expansion than orthodontic and dental stone. The FBG technique can be a good tool for dentists to better manipulate a material and predict how it will behave *in vivo*.

A research group in Portugal has employed FBGs to measure strains at the surface of an implanted cadaveric mandible caused by impact loads. Although they had previously used SGs in a few studies pertaining to the mandible [71,72], they studied the potential use of FBG as a biomechanical sensor [73]. In one of their studies [74], they measured the dynamic strains at the mandible surface induced by impact loads. A standardized dental implant was implanted in a dried human cadaveric mandible embedded in a resin support, and an FBG was placed on the outer surface of the mandible near the canine tooth position in a direction parallel to the implant longitudinal axis. The impact loads were generated by striking the implant with a steel mass (52 g) dropped inside a steel tube. The obtained results agreed with those previously reported studies using SGs, showing the ability of FBG sensors to measure dynamic strain variations, with precision, in complex biomechanical systems.

**Figure 9.**
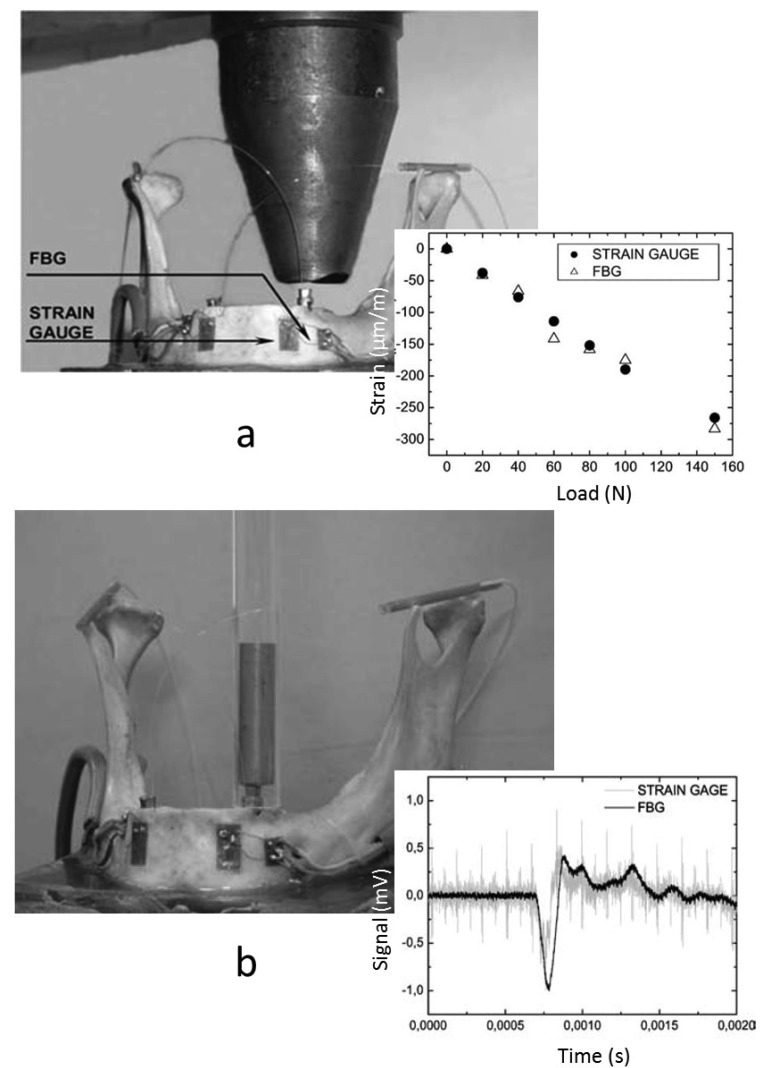
(**a**) The set-up of the step loads. The FBGs and SGs attached to a cadaveric mandible. The inset illustrating the strains measured by both techniques. (**b**) The set-up of the measurement of dynamic strains and the inset showing the FBG versus SGs strains in time-domain [75].

In a subsequent work by the same group [75], they have measured the strains at the mandible surface due to both static and impact loads, using FBGs and SGs, and two standardized implants were implanted in the cadaveric mandible used in the previous study. Two uncoated FBGs and two uniaxial SGs were placed on the mandible surface in the longitudinal axis of the implants (One FBG and SG near each implant), as shown in Figure 9. For the static loads, the implant was step loaded up to a maximum of 160 N, and strains at the surface were recorded by each sensor, FBG and SG. The impact loads were generated in a manner similar to that used in the previous study [74] which was done by striking the implant with 52 g a steel mass. The results of both types of sensors were compared, showing very similar response when both static and dynamic loads were applied. The FBG sensor exhibited better sensitivity with small strains compared to SG, showing its feasibility to be used as an alternative method for measuring strains in dental biomechanical applications.

FBGs can also be used to study model mandibles that replicate the mechanical properties of human bone. A synthetic model was used to study the strain distribution over the mandible branch and articulation caused by the action of the muscles [76]. Four FBGs were glued at the outer surface of the mandible and the strain patterns were assessed for different load configurations including the forces of the masseter and temporal muscles and occlusion loads on different teeth. The same device was modeled by Finite Element (FE) method to determine the strain at the mandible surface. The strains obtained from the FBGs were correlated to identical ones obtained with a numerical FE model. The comparison of the results is considered satisfactory. For symmetric loading the strains measured were identical. For the non-symmetric loading, the FE boundary conditions must be simulated more accordingly. Such results validate numerical methods used in the design of mandible and condoyle prosthesis.

Measuring strains in the mandible bone helps predict the load transfer from the implant to the surrounding bone, leading to design novel dental implants that minimize the strains induced in the mandible. Macro-models of dental implants were studied with FBG sensors inserted in real bone using samples from bovine femur [77,78]. Three types of dental implants; metallic, polymeric and polymer coated metal implants, were compared in order to study the load transfer from the implant to the bone. The FBGs were inserted into the bone in a direction parallel to the implant axis and the static and dynamic strains were recorded as a function of loads. The results show that polymer coated implants can be more appropriate due to better load transfer. FEM analysis can be performed in order to compare and validate the experimental results.

FBG sensors have been used to measure the forces applied onto the surface of teeth and the consequent displacement caused by the orthodontic systems. Low magnitude applied forces caused by orthodontic systems are considered more effective for moving the teeth than impulsive loads. Therefore, Milczeswki *et al.* [79] investigated the forces applied onto the surface of an incisor by orthodontic system using FBG technology. The experiments were performed in an artificial maxilla with dental wax and metal teeth and the teeth were instrumented with a fixed orthodontic appliance. An FBG imprinted in a HiBi fiber was placed between the incisor surface and the orthodontic bracket, and loads were applied representing the auxiliary appliance. The anterior-posterior force and displacement of the tooth movement were measured, showing that the small size and the high sensitivity of the FBG sensor allow for the determination of forces applied orthogonally to the surface of the teeth. This technique is promising and can be used in the other types of orthodontic applications for *in vivo* experiments.

Recently, Milczewski *et al.* [80] went a step further and applied a similar approach using FBGs to study the magnitude and location of forces at the teeth roots and maxilla bone during applying load by orthodontic appliance. A maxilla model was instrumented with FBGs, three pairs of FBGs positioned along three artificial teeth roots, immersed in the elastomeric material, and the other four multiplexed fiber sensors placed transversally along the teeth apex. Preconditioning tests were performed to evaluate the sensitivity of the Bragg gratings *in-situ*. After the activation of the orthodontic appliance, the teeth were loaded and the strain on the maxillary bone and dentition was monitored showing a linear relationship between the strain and the applied load in the incisor, canine and molar teeth. This also can show the transference of the forces to the teeth and finally to the internal maxillary structures.

**Figure 10.**
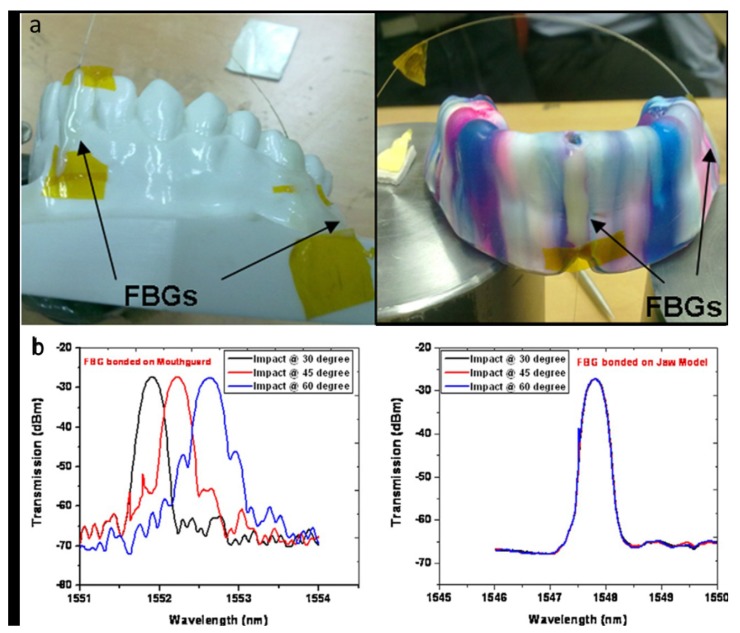
(**a**) The jaw model and mouthguard with FBGs. (**b**) The response of ball impact portraying no changes for FBG bonded on jaw model (**b right**) as compared to FBG bonded on the mouthguard (**b left**) [3,81].

In a recent (2011) study by Tiwari *et al.* [81], FBG sensors were utilized to monitor the impact absorption capability of mouthguards. To assess the mouthguard efficiency when subjected to impact loads, the strain transfer to the teeth through a custom-made mouthguard was studied. Two sets of FBGs were used, one placed at the anterior region and another at the posterior region. Each set has two FBGs; one was attached to the jaw model and the other on the mouthguard surface at parallel locations so that both FBGs are simultaneously affected by the impact (Figure 10(a)). Impact was made using a pendulum device with interchangeable round objects, *i.e.*, cricket ball, hockey ball, and steel ball, and strain induced due to each impact was determined from the FBG wavelength shifts. The wavelength shifts for the FBG bonded on the jaw model exhibited much lower than the shifts for the FBG bonded on the mouthguard (Figure 10(b)), indicating that most of the impact energy is absorbed by the mouthguard. This study can help design better custom-made mouthguards for different malocclusion, as the FBG technique can give strain not only at the point of impact but at other vulnerable points also in a single hit.

### 3.6. Chest Wall Deformation

The monitoring of chest wall movement during respiration is crucial in understanding lung biomechanics. The chest displacement due to respiration normally ranges from 4 to 12 mm [82,83]. Sensors composed of a bent optic fiber for the measurement of respiratory chest circumference changes had been previously developed by Babchenko *et al.* [84]. This sensor was composed of a bent optic fiber connected to a chest belt so that its radius of curvature would change during respiration due to changes in the respiratory chest circumference and the light transmission was measured through the bent fibre providing information on the curvature changes. However, this method was replaced with FBG technique which was first reported by Wehrle *et al.* in 2001 [85]. In this study, the measurement of dynamic strain on human chest during respiration was developed and tested. The FBGs were embedded into a chest deformable strap which was strained in accordance with the deformation of chest. This sensor showed its ability to detect deformation in the thoracic cage during respiration, indicating the respiratory frequency spectrum. It can also be used to trigger a device for inducing electrically assisted ventilation, when one lung is to be excited in phase with the other working lung, or to monitor high frequency oscillatory ventilation. Moreover, the FBG sensors showed their capability to measure the muscle movement in the chest.

It is no longer strange to combine electronics and textiles into a single structure to produce electronic textiles (e-textiles) [86]. Recently, a new project supported by the Europe Union and called “Optical Fibre Sensors Embedded into technical Textile for Healthcare (OFSETH)” was launched [87]. The aim of OFSETH is to take advantage of pure optical sensing technologies for extending the capabilities of medical technical textiles for wearable health monitoring [88]. These wearable smart textiles can be used to simultaneously monitor the vital physiological parameters such as respiratory movements, cardiac activity, pulse oximetry, and temperature of the body. They exhibit advantageous properties over other standard (non-optical) monitoring techniques, especially for monitoring the anesthetized patients under Medical Resonance Imaging (MRI) [89–91].

The OFSETH project proposed three optical sensing techniques for monitoring the elongation in textile fabrics worn by patients: the bending loss sensor, the FBG sensor and the optical time domain reflectometry sensor. Grillet *et al.* [87] have reported on these three different techniques, all smart textiles embedded. In this study, only the elongation of the abdominal circumstances during ventilation was measured unlike the study by Wehrle *et al.* [85] which measured the thorax movement instead. The textile was fixed to a load cell and a movable platform by clamping blocks to investigate the stretching capabilities of the smart textile and the effective strain on the optical fiber was then monitored during the stretching experiment by continuously recording the shifts in Bragg wavelength. The FBG sensor performance during cyclic strains mimicking those normally induced by chest movements was also tested to assess the suitability of this proposed design. The results showed that these three techniques are able to sense the textile elongations between 0% and 3% and the stretching properties of the textile was maintained during the measurement to ensure the patient comfort. Moreover, the FBG sensor embedded in the textile is able to sense weak breathing movements with a better resolution.

**Figure 11.**
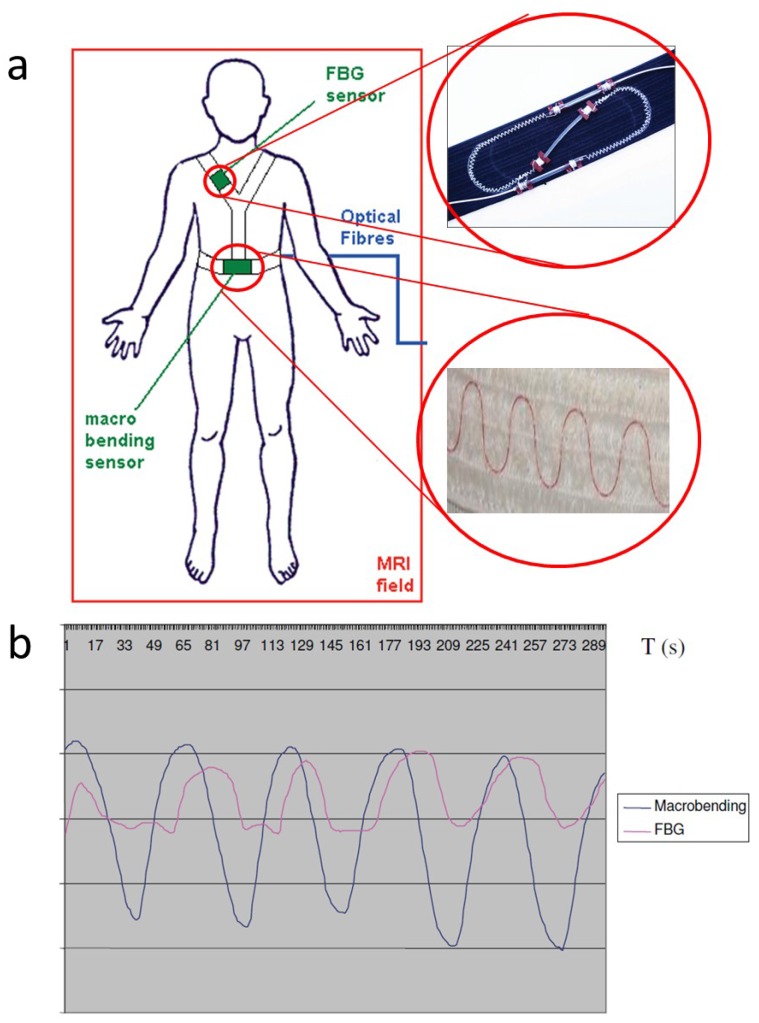
(**a**) The optical fibers-textile integration, illustrating the optimal positions of macro-bending and FBG sensors. (**b**) The signals of both the abdominal and thoracic movements using both types of sensors [92,93].

Another work by Jonckheere *et al.* [92] reported on only two pure optical sensing techniques—macro-bending sensor and FBG sensor—embedded into textile fabrics to measure the elongation of the technical textile caused by both the thoracic and abdominal movements during ventilation. The aim is to ensure the proper position of the optical sensors in order for minimizing parasitic effects such as mechanical strains and optical losses, and to convert all the textile elongation into a mechanical force that will be transferred to the sensors. The typical positions of these two sensors are shown in Figure 11(a). The combination of these two designs produces a device allowing for continuous monitoring of both thoracic and abdominal movements. The two designs showed their capability to measure the textile elongation between 0% to 3%. The thoracic and abdominal movements signals obtained (Figure 11(b)) can indicate the respiratory system efficiency and breathing frequency spectrum. This shows that optical sensors can be the golden solution for the measurement of vital signs of patients during MRI examination as it shows immunity to electromagnetic radiations. However, these sensors were then developed to be able to measure both the thoracic and abdominal movements with increased elongation between up to 5% and to be connected to a hard monitor or to a mobile device [93].

The FBG sensors previously developed to measure the chest strain due to breathing has been continuously advanced, allowing for detecting both respiratory motions and cardiac frequency simultaneously by using a single FBG, rather than using the macro-bending loss effect of an optical fiber [94–96]. Although FBG sensors have been used to detect heart sound to evaluate the heart muscles function, which reflect the biomechanics of the heart and great vessels effectively, it was based on an approach enabling the heartbeat measurement only [97]. However, a developed system compatible with different people to measure both components, the respiratory motions and heart rate, using a single FBG sensor was proposed, introducing different measurement methods and sites [98–100]. Silva *et al.* [98] have developed a FBG sensor embedded in a PVC foil to enable a large sensitive area and enhance the sensitivity. This sensor can be attached to and removed from the chest site via Velcro or even be worn by the subject. A signal processing system was used to filter out the cardiac and respiratory frequencies. A commercial device was utilized for referencing and the optical fibre system was tested on 12 healthy subjects who participated in this study. This system was reliable and found an application niche in MRI environment, where no other types of sensors than optical ones can be used due to EMI.

Wireless technology is widely used in biomedical and life monitoring, such as the monitoring of breath motion. A great effort has been conducted throughout the World to develop mobile approaches for chest movements monitoring. The use of optical fibers embedded into textiles for mobile respiratory motion detection was first reported by D'Angelo *et al.* [101]. This mobile system involves a T-shirt with an integrated fibre sensor and a portable monitoring unit, and wirelessly connected to a PC to enable the data analysis and visualization. The authors used the bending of the optical fibers to recognize and analyse the respiration of the wearer's chest. However, a novel FBG with a wireless-based approach was introduced by Xiaobin *et al.* [102]. In this study, the principle of “Four States Method with Combined Modulation” was used in the FBG demodulation, so that this new method could have the features of small size, high integrality and portability. This proposed system can monitor the respiratory movements with a sensitivity of about 41 με. Furthermore, an alarm signal can be transmitted wirelessly to a remote server when abnormal event occurs. These wireless approaches satisfy the requirements to measure chest deformation during breathing, and, at the same time, provide the convenience to the patients.

### 3.7. Human Machine Interfaces

Investigation of the pressure ulcer in the Human Machine Interfaces (HMI) is critical to understand the mechanism of the body movement. An embedded array of FBG sensors can be employed in diabetic foot/shoe interface, clinical beds, prosthetic sockets and wheelchair seating systems, targeting pressure ulcer development and wound treatment [103]. Recently, the IASiS project was established, aiming at bringing optical fiber sensing in rehabilitation engineering, especially at the area of HMI research [104,105].

#### 3.7.1. Insole Sensors

Properly-designed temperature-independent FBG sensors can be used to map the foot pressure distribution of diabetic patient's plantar surface through the gait cycle, so as to understand the gait mechanism and diagnose the foot pathology. This helps orthopedic surgeons and doctors determine the actual normal forces exerted at the key pressure points under the patients' foot, the total applied force and the center of pressure. Conventional insole pressure measurement systems employ capacitive [106], conductive [107], piezoelectric [108], resistive [109], and magneto-resistive [110,111] types of transducers which show limited performance and require a heavy shielding, causing an increase in the cost and size of the sensors significantly [112]. The fibre-optic bend loss sensors were also investigated [113], showing good reliability and acceptable accuracy. In contrast to the above systems, FBG pressure sensors employ an all-passive dielectric approach offering many advantageous properties such as electrical isolation from the patients, immunity to EMI, light weight and compactness [4]. In addition, they exhibit higher sensitivity and the capability to be multiplexed and integrated.

A research group based in Singapore has investigated the potential use of FBG-based sensors for monitoring the foot pressure of diabetic patients [112]. Five FBGs with five different wavelengths were fabricated and spatially placed along a single strand of grating fiber in order to monitor different pressure points under foot. They were sandwiched with eight layers of carbon/epoxy laminates on top and two layers at the bottom to form the foot pressure sensor which can be cut into the shape of a footpad. Clinical tests were conducted on a 26-year-old male patient with a body weight of 60 kg. A comparison between the pressure distribution of a normal standing gait and an abnormal standing gait on the right foot were conducted. This sensor shows a better performance than the conventional foot pressure sensing systems in terms of sensitivity, cost and compactness.

Other researchers have also used FBG foot sensors to study the biomechanics of human balancing on a vibrating floor [114]. Three multiplexed FBG sensors were used at the bottom of each foot to provide information on areas where the pressure would be most concentrated. The pressure distribution over different parts of the foot was obtained at different frequencies and different distances from the vibrating source. All observations showed that most of movements occur on the right foot especially around the heel area, which—in turn—show that the right foot is more sensitive to vibration which is consistent with the natural behavior of a normal person whose one leg is more agile and sensitive than the other. These findings show great potential for the application of FBG foot sensors to study the reaction of the human being subjected to floor vibration. Furthermore, the system shows its robustness and ability to work under harsh conditions.

FBG sensors also hold great potential for measuring the in-shoe shear stresses. Although several methods for the shear measurements had been developed [110,111,115,116], they showed common drawbacks which are the relatively large size of the sensors and difficulty in interrogating many sensors at a time to achieve quasi-distributed sensing. In contrast, FBG sensing is attractive method due to its small size and multiplexing capability. Koulaxouzidis *et al.* [117] investigated the feasibility of FBG sensors to simultaneously measure the in-shoe normal and shear stresses to understand the planter ulcer development after the onset of diabetes. With special configuration, three FBGs were embedded in a block of an epoxy elastomer to be capable of measuring the vertical stresses as well as the magnitude and direction of the shear stresses on its top surface. The experimental results showed the applicability of this design and the effectiveness of the readout system. The good repeatability and high resolution in both directions and its small size are key advantages as well.

#### 3.7.2. Amputee Sockets

There is a potential use of 2D or 3D FBG sensor structures to measure the pressure and shear stresses in the interface between amputee's socket and stump. Since the mid-1960s, researchers have proposed a variety of measurement systems such as ESG based transducers [118–120], F-socket transducer arrays [121], and FEA [122–124] in order to understand the pressure distribution in this interface, leading to fabrication of amputee sockets that prevent discomfort and provide satisfactory results. Despite these technological advances in the existing socket design and the measurement systems, many amputees still complain that their prosthesis causes complications such as edema, pressure ulcers, skin irritation and dermatitis [125]. However, the use of FBG sensors could show more accurate results which can assist in the development of better socket designs that help amputees live normal day-to-day lives with minimal complications. Kanellos *et al.* [126] developed a flexible 2D FBG pressure sensing sheet suitable for biomechanical applications such as amputee socket/stump interface. The sensor mechanical properties matched human skin behaviour and its operational performance exhibited a maximum fractional pressure sensitivity of 12 MPa-1 with a spatial resolution of 1 × 1 cm^2^ and demonstrated no hysteresis and real-time operation. These attractive properties meet the requirements of HMI pressure measurements including amputee sockets.

In the EC FP7 project “IASiS” studies have introduced an FBG based 2D sensing surface that could continuously monitor the pressure and potentially shear applied to below-knee stump/socket interface [127,128]. Pleros *et al.* [103] also demonstrated the evolutionary road towards smart FBG-based HMI structures for pressure ulcer monitoring in amputee socket HMI systems. They addressed the pressure and shear by using arrays of multiplexed FBG sensors by introducing two parallel layers of 2D sensing FBG meshes allowing for shear sensing by correlating the differences in the strain-induced deformation of the two FBG mesh layers. These two mesh layers form a 3D sensor structure that could measure both the pressure and shear stress at a time. This developed sensor is suited to be utilized in HMIs structures such as stump/socket interfaces.

A recent study by Kanellos, Tsiokos, Pleros, Childs and Pissadakis [105] presented the optimal design of FBG sensing pad that exhibits enhanced durability and higher sensitivity and is suitable for applications in HMI surfaces such as amputee sockets, medical beds and wheelchairs. They investigated three case studies: fibre embodiment depth (centre and top position of pad cross-section), pad thickness (2 and 3 mm) and fibre type (hydrogenated SMF-28 and non-hydrogenated GF1B). In the first study case, two hydrogenated SMF-28 fibre FBG sensors were embedded in a 2 mm thick PDMS sheet in the centre and top position of the cross-section. A series of vertical loads were applied using a gauge test stage. The results revealed a 50% increased sensitivity and a 40% increased durability for the FBG positioned in the center of the PDMS layer. In the second case study, they compared the sensitivity and durability of hydrogenated SMF-28 fibre FBG sensors embedded in the center of a 2 mm and 3 mm thick PDMS sheets respectively. After the test, it was found out that the sensor sensitivity was increased with 50% and a marginal increase in the durability for the thicker (3 mm) sensor pad. Finally they compared the sensitivity of two FBGs inscribed in two different fiber types (hydrogenated SMF-28 and non-hydrogenated GF1B). The results exhibited 20% increased sensitivity for the hydrogenated fiber and a 40% decrease in its dynamic range when compared to the non-hydrogenated fiber. It is clear that the optimum sensor pad layout enhancing durability and preserving required sensitivity and can be successfully implemented in HMI applications is when the FBG is written in a non-hydrogenated fiber and embedded at the centre of the thicker (3 mm) PDMS sheet.

**Figure 12.**
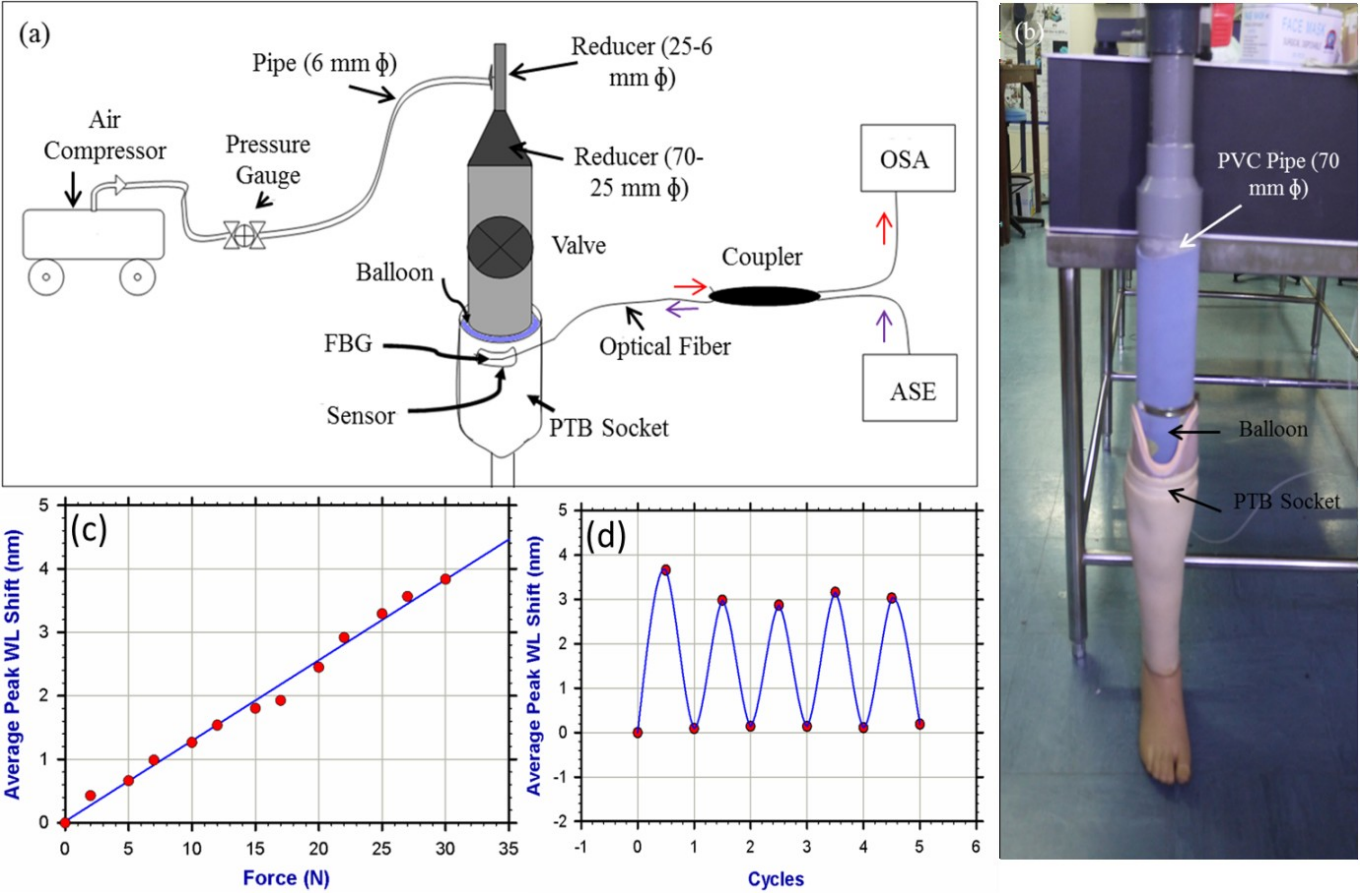
(**a**) A simple schematic diagram of the in situ test. It shows the sensor while connected to OSA and subjected to loads. (**b**) Depicts the image of the set up. (**c**) The calibration of FBG sensor showing the FBG peak wavelength shift versus the applied force. (**d**) Cyclic change in pressure from minimum (0 Pa) to 35 kPa was performed to test the repeatability and reliability of the sensor. The solid line is for indicative purpose only.

The previous researches proposed sensors that could be suitable for simultaneous measurement of the normal and shear stresses at the amputee socket/stump interfaces. To date, full-scale implementation of these sensors in this area of research has not been established yet. In a project entitled “Synthetic Prosthetic Socket through Stump—Liner Interfacial Stresses Measurement” which supported by the Ministry of Higher Education—Malaysia [129], our research group and in collaboration with Photonics Research Group [130] in the department of electrical engineering at University of Malaya aims to establish investigative studies on the capability of the optical FBG sensors to measure socket/stump interface pressures, leading to the development of a novel interfacial stresses measuring system that can also be applied over surfaces of irregular shapes. Currently, we are focusing on designing an FBG sensor that can be used in transtibial amputee socket/stump interfaces and capable to measure the normal and shear stresses simultaneously. We started to implement a single FBG embedded in a polymeric material and bonded at the patellar tendon bar area in order to measure the pressure at this most tolerant area within the amputee socket. This sensor was tested in real-time by inserting a heavy duty balloon into the socket and inflating it via an air compressor in order for simulating the pressure applied by real stump and for examining the sensor sensitivity and repeatability *in-situ* (Figure 12). The results exhibited that the sensor has a good sensitivity and acceptable hysteresis. We are currently working hard to involve subjects in our experiments in order to clinically validate this design. Plans to implement experiments to map the pressure as well as the shear stress over the whole inner socket wall and compare this system to the conventional ones are currently in progress.

#### 3.7.3. Bedridden Patients

The utilization of FBG sensors in HMI systems was extended to their use in hospitals and nursing homes to automatically monitor the behaviour of clinical bedridden patients and their vital signs. When paralysed patients lying on beds stay unmoved for long time, bedsores generation occurs. Currently, monitoring of bedridden patients is usually handled by caregivers and professionals who must manually conduct periodic checks. This practice is tedious and time consuming and the occurrence of delayed response is highly expected. Therefore, development of multiplexed FBG pressure sensors which can be strategically distributed on the top surface of clinical beds is highly recommended to continuously monitor the patients sleeping and sitting pattern and to alert the nurse to help move the patient's body when unmoved for long time, in order to prevent bedsore generation.

**Figure 13.**
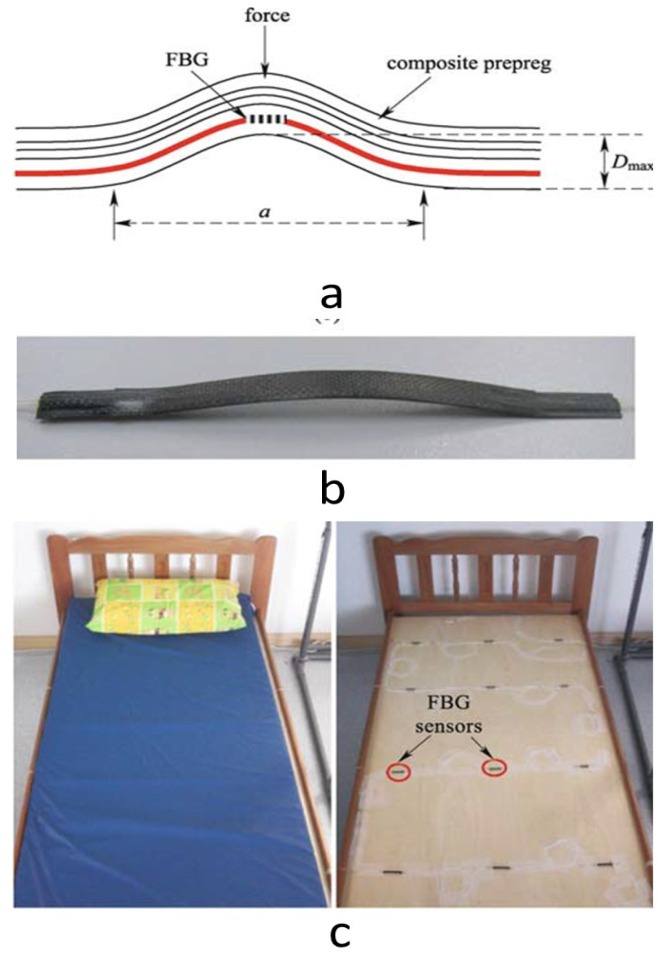
(**a**) A drawing illustrating the FBG embedded in a composite laminates. (**b**) Arc-shaped sensor. (**c**) A 3 × 4 array of FBGs deployed on bed [131].

Hao *et al.* [131] have developed a smart multi-functional FBG sensor system which can map out the pressure points beneath the patients and monitor the respiratory rates and other parameters as well. In this study, twelve FBG sensors embedded in arc-shaped carbon fibre reinforced plastic material were cascaded along a single fibre and mounted on the surface of the bed to form 3 × 4 matrix array, and the bed was then covered by the usual mattress (Figure 13). A sample group of 10 subjects were undergone trials in the laboratory and the system captured and displayed the body movement and respiration pattern. This system showed promising effectiveness and ability to work. Such systems show advantageous features if compared with the current commercial systems demanding the patients to wear specialized probes which introduce some level of discomfort. However, more studies are recommended to advance these systems to be able to accurately capture most of the vital signs that help practitioners observe the bedridden patients properly.

### 3.8. Tendons and Ligaments

Accurate and precise measurements of ligament and tendon biomechanics in living humans are vitally essential in better understanding function and injury and in optimizing treatment [132]. FBG sensors have been used to study the biomechanical properties of tendons and ligaments and shown encouraging results. Vilimek proposed the use of FBG sensors to investigate the *in vitro* biomechanical properties of porcine tendons using a tensile machine (MTS Minibionix) in vertical orientation [133]. The FBG sensor was crosswise included by grouting point into the tendon whose ends were attached to the actuator using the freeze clamping technique. The sensor was repeatedly loaded and unloaded in steps using the MTS and the measurement was run until the tendon was destructed. In all of the trials, the measured forces by MTS and measured signals from FBG sensor give the same proportional changes and it is possible to estimate the loading force. The FBG sensors were more sensitive and accurate than other fibre-optic methods based on decrement of light properties during pressing change of cross section size. This approach is suitable for *in vivo* measurement of a musculotendon forces in humans for validation the muscle force computational models because the injury after application is minor.

Ren *et al.* proposed a FBG-based displacement sensor for monitoring strains developed in tendons and ligaments in different postures and in locomotion, and then compared the obtained results to those obtained using traditional camera displacement sensors [134]. The FBG was embedded in a micro-shape-memory-alloy tube which was then placed on an Achilles tendon surface extracted from human cadaver and the whole system was mounted on a material testing device for loading test. After sensor calibration in the laboratory, the performance of the FBG sensors and traditional camera displacement sensors was compared. Additional experiments were performed in cadaveric knees to assess the feasibility of the FBG sensors to measure ligament deformation in a variety of simulated postures. The results demonstrate that the proposed FBG sensor is a highly accurate, easily implantable, and minimally invasive method of measuring tendon and ligament displacement as compared to the traditional measurement methods.

### 3.9. Human Body kinematics

The monitoring of different human body kinematics and the analysis of posture and gesture are crucial in bioengineering and rehabilitation. It aims at improving athletic performance in competitions and at evaluating frequently the patients undergoing therapeutic treatments to know the efficiency of the prescribed therapy. Several systems have been previously proposed for monitoring human body kinematics [135–139], however, they all are not able to withstand some specific environments like chemical solutions, electromagnetic noise and high temperature, among other [140].

**Figure 14.**
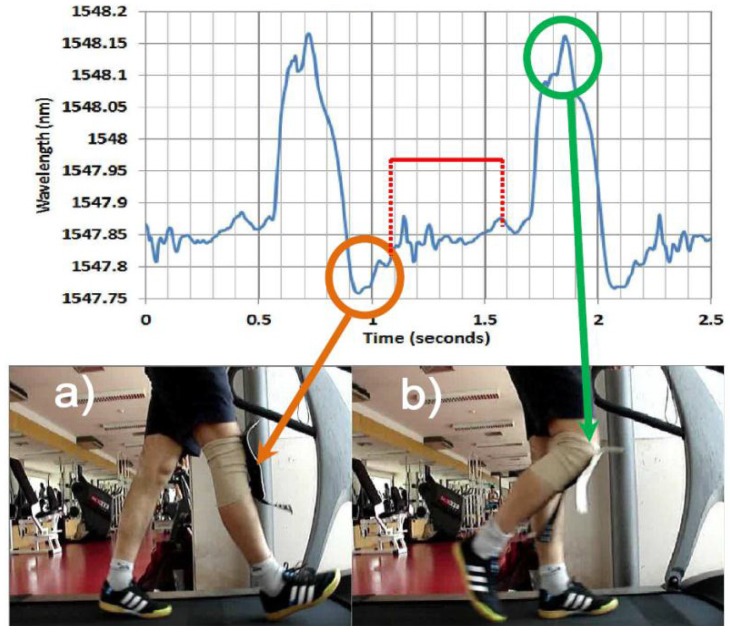
The wavelength values for a walking at 4 km/h. (**a**) and (**b**) show the minimum and maximum deflections of the FBG, respectively [147].

Recently, a research group based in Portugal has proposed a smart skin polymeric foil based on FBG sensors which is suitable for the measurement of body kinematics [141–146]. Rocha *et al.* [147] have validated this wearable system by monitoring maximum knee flexion and extension and all the movements in-between during a full period of human gait. A single FBG was placed at the center of the knee joint and embedded in a rectangular-shaped structure that is composed by three layers of flexible and stretchable PVC materials. This sensing structure can be easily attached to/removed from an elastic knee band by using small metallic pressure buttons to ensure that the FBG is capable of detecting only the flexion and extension as the subject moves around. The subject underwent different types of running and walking on a commercially-available treadmill in order to assess the reliability and consistency of this system and the movements of knee joints were being measured as a function of the FBG wavelength, and, at the same time, with video recording for comparison purposes. The results show that the stage where the maximum flexion/extension in the knee joint, and also on the FBG, represents the maximum/minimum wavelength values as depicted in Figure 14(a,b). This sensing structure shows a good sensitivity to accurately measure the knee flexion/extension during the walking and running tests and is able to be used for any joint in human body. This FBG-based PVC foil opens up the road for many applications in biomechanics and other arenas.

**Figure 15.**
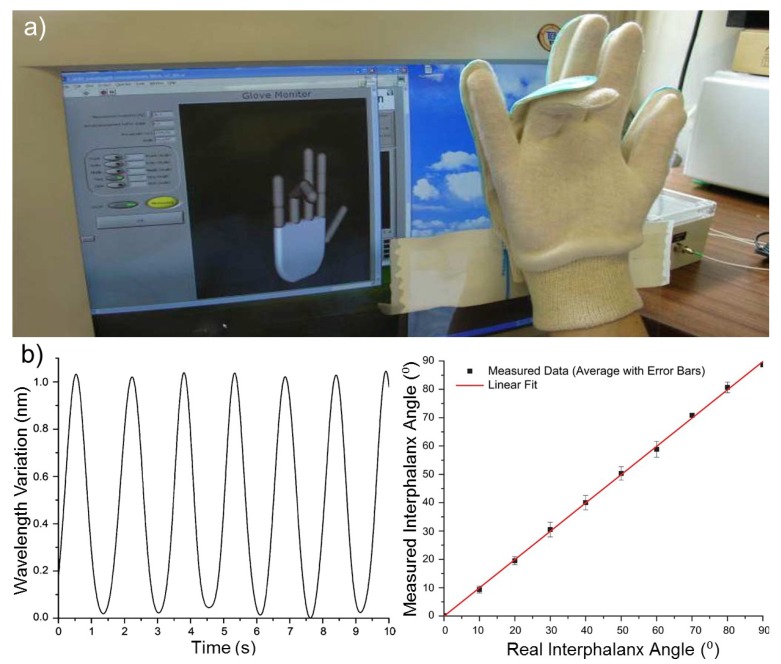
(**a**) A hand-shaped PVC foil accommodating 14 FBGS and sewed to the upper face of a standard glove, allowing for the visualization of the hand movement in real-time on a PC. (**b**) (left) The response of ring finger FBG sensor for opening and closing hand movements. (right) A curve comparing between real and measured angles, illustrating the system accuracy [153].

The monitoring of hand gesture and posture of brain stroke patients is of interest in rehabilitation engineering in order to assess the hand ability to perform action in a proper manner during therapeutic sessions. Several wearable glove systems using different approaches have been proposed [148–152], but demonstrated some concerns [153]. Silva *et al.* have designed a wearable sensing glove based on FBGs in order to enable the measurement of angles between the finger phalanxes and to overcome the limitations presented by other previously reported methods [153]. Fourteen FBGs accommodated in a single optical fiber were strategically placed in a curvilinear layout and embedded in the middle layer of a flexible PVC foil cut into the hand shape so that each FBG would be positioned over each of the 14 phalanx joints present in a single hand. Thereafter this customized hand-shaped sensing foil was sewed to the upper face of a standard fabric glove (Figure15(a)) to guarantee that every FBG is positioned in its intended place throughout the experiments. Tests to evaluate the FBGs performance and functionality according to hand flexion and extension were performed. The sensor exhibited a sinusoid-like response (Figure 15(b), left) where the positive and negative limits represent the hand closing and opening actions, respectively. It is possible to estimate the strength from which the 1 nm wavelength shift resulted from an applied load of 7.8 N, leading to retrieve information about joint angles. A comparison between the real and measured angles produced a linear fit, representing the system accuracy with R-square of 0.99929 and a slope of 0.99869 (Figure 15(b), right). In the second part of this study, a hand motion capture system based in a 3-D virtual model of the hand was built (Figure15(a)), allowing for the visualization of the hand movement in real-time on a PC which can be used to provide information about the hand angles, strength, and movement range. This wearable system could have a great potential in physical therapy applications, in particular for hand-impaired people, and when studying human kinematics, among others.

## 4. Conclusions

It is clearly evident from the literature that FBGs can be successfully employed in a wide range of applications in biomechanics and rehabilitation engineering. The reason of this is because FBGs have a number of distinguishing advantages, over other traditional systems, such as their small size, chemical inertness, light weight, biocompatibility and multiplexing capability. Although FBGs have proven their feasibility and superiority over existing technologies, they have not been commercialized yet in the field of biomechanics and rehabilitation. With the rapid developments in this field and the need to successfully implement FBG sensors, a collaborative work involving medical doctors, experts in fiber-optics and programming engineers must take place in order to establish the practicality of this technology, taking into consideration that FBG sensors must be user-friendly, cheap in cost, and easy-to-use products so as to satisfy the requirements of medical doctors and end users as well. Very recently, portable interrogation systems with high-speed data acquisition (up to 5,000 Hz) were made available, leading to broadened research areas and finding new niche applications. This article is a comprehensive review that covers all the possible uses of FBG sensors and the ongoing researches worldwide in biomechanics and rehabilitation engineering. It also exposes the superior advantages of FBGs when compared to the other existing technologies. It is believed that FBG sensors will undoubtedly have a major role in the future and provide effective solutions for a wide variety of applications in biomechanics and rehabilitation engineering.
